# Evaluation of Retinal Nerve Fiber Layer and Ganglion Cell Complex in Patients with Optic Neuritis or Neuromyelitis Optica Spectrum Disorders Using Optical Coherence Tomography in a Chinese Cohort

**DOI:** 10.1155/2015/832784

**Published:** 2015-11-15

**Authors:** Guohong Tian, Zhenxin Li, Guixian Zhao, Chaoyi Feng, Mengwei Li, Yongheng Huang, Xinghuai Sun

**Affiliations:** ^1^Department of Ophthalmology, Eye, Ear, Nose, and Throat Hospital, Fudan University, Shanghai 200031, China; ^2^Department of Neurology, Huashan Hospital, Fudan University, Shanghai 200040, China

## Abstract

We evaluate a cohort of optic neuritis and neuromyelitis optica (NMO) spectrum disorders patients in a territory hospital in China. The peripapillary retinal nerve fiber layer (RNFL) and macular ganglion cell complex (GCC) were measured using spectral-domain OCT after 6 months of acute onset. The results showed that both the peripapillary RNFL and macular GCC were significantly thinner in all optic neuritis subtypes compared to controls. In addition, the recurrent optic neuritis and NMO groups showed more severe damage on the RNFL and GCC pattern.

## 1. Introduction

Acute optic neuritis may be the first manifestation of both multiple sclerosis (MS) and neuromyelitis optica (NMO), or some unknown etiology of disorders [1, 2]. In Chinese, the demographic and clinical features of optic neuritis spectrum disorder are less well-defined than that in Caucasus [3–5].

During the past few years, numerous studies showed that peripapillary RNFL and macular thickness analysis may be used to detect axonal loss in optic neuritis, neuromyelitis optica, and other forms of chronic relapsing optic neuritis [6–9]. In addition, it has been suggested that OCT abnormalities can help differentiate MS from NMO on the severity of axonal loss [10–14].

Due to the ethnic differences, optic neuritis in China shows more atypical features than those in western countries and the prognosis is not clearly described. The propose of this study was to evaluate the thickness of the RNFL and macular GCC using SD-OCT in different forms of optic neuritis in a cohort of Chinese patients and compare the pattern of damage in MS-ON, NMO-ON, and R-ON group.

## 2. Materials and Methods

### 2.1. Patients

The current study was a cross-sectional study. Patients who presented with acute optic neuritis in Neuroophthalmology Division in Eye Ear Nose and Throat hospital, Fudan University, Shanghai, between May 2013 and January 2014, were recruited. Paper consent forms were obtained for participation through a study protocol that was approved by the hospital institutional review board. All patients had their diagnosis confirmed by referred neurologists and neuroophthalmologists. After thorough ancillary tests and at least one-year of follow-up, patients were divided into 3 groups for evaluating the involved eye: MS-ON, R-ON, and NMO-ON.

MS-ON group patients included typical acute demyelinating optic neuritis with brain lesions fulfilling the revised McDonald criteria or clinical isolate syndrome (CIS) [15, 16]. Recurrent isolated optic neuritis (R-ON) was defined as unilateral or bilateral recurrence affecting optic nerves in patients whose clinical evidence showed no other brain lesion and seronegative AQP4-Ab. A diagnosis of NMO-ON was given to patients who met established diagnostic criteria for NMO or NMO spectrum disorders (NMO-SD) published by Wingerchuk et al. [17]. The new onset eyes in three groups were included for OCT evaluating. As for acute bilateral involved patients, only one affected eye was randomly chosen for OCT evaluation. As for R-ON patients, the new attack eye was evaluated. All enrolled patients underwent the routine blood test including the infectious panel, the rheumatology panels. All patients underwent serum AQP4 antibody test in neurobiology laboratory using the ELISA methods (kit from ElisaRSR AQP4-Ab, RSR limited, UK). Neuroimaging was required to confirm the acute attack of the optic neuritis, evaluate brain demyelinating, and exclude the compressive optic neuropathy and anterior ischemic optic neuropathy.

Exclusion criteria included patients with pathologic myopia with spherical equivalent of the refractive error >6.0 diopters, a previous history of ocular disease (including macular degeneration, diabetic retinopathy, uveitis, and glaucoma), and neurodegenerative conditions that could impact OCT testing results (Parkinson's disease, Alzheimer's disease), and subjects with poor vision having difficulty maintaining fixation were excluded from analyses.

The group of control was recruited from volunteers of hospital staffs and patient's companions at the time duration of the follow-up. Inclusion criteria included best-corrected visual acuity of at least 20/20, spherical equivalent of the refractive error <6.0 diopters (highly myopic), without presence of any ophthalmic or neurological diseases known to affect RNFL thickness. One eye was randomly chosen for evaluation.

### 2.2. Optical Coherence Tomography

Spectral-domain optical coherence tomography (SD-OCT) was performed using 3D Disc, ONH, GCC protocols provided by the RTVue-100 4.0.7.5 version (Optovue Inc, Fremont, CA). An internal fixation target was used to improve reproducibility. Scan was accepted only if the images with a signal strength index were greater than 35. The peripapillary RNFL thickness was measured automatically using a RNFL 3.45 scan mode, where 4 circular scans (1024 A-scans/scan) acquired 3.45 mm from the center of the optic disc. The RNFL was divided into temporal (316°–45°), superior (46°–135°), nasal (136°–225°), and inferior (226°–315°) quadrants. A RNFL progression analysis is also available for follow-up.

The GCC scan technique provides inner retinal thickness values which consist of ganglion cell layer (GCL) and inner plexiform layer (IPL). Scan mode for GCC analysis, which acquires 14 928A scans over a 7 mm square area in 0.58 seconds with 15 vertical scans collected at 0.5-mm intervals. The center of the scan was shifted 1.0 mm temporally to improve sampling of the temporal periphery. The GCC within the central 6 mm diameter area of the macular was calculated. All the data were measured and collected in acute optic neuritis patients 6 month after attack.

### 2.3. Statistical Analysis

Demographic variants were described and compared by ANOVA test (numeration variables) if the variance was homogeneity or chi-square test (categorical variables). The peripapillary RNFL data measured according to 4 quadrants was analyzed using repeat measurement analysis of variance due to the correlation intereye within patient. The difference between each group was statistic and compared with controls. GCC values were analyzed by independent-samples *t* test between groups. A *P* value of less than 0.05 was considered statistically significant. All analyses were conducted using IBM SPSS statistics for windows, Version 19.0 (IBM Corp, Chicago, USA).

## 3. Results

### 3.1. Demographics

A total of 118 patients, including MS-ON (*n* = 62), R-ON (*n* = 19), NMO-ON (*n* = 37), and 68 healthy controls were evaluated. The demographic and clinical characteristics are summarized in Table 1. Among the MS-ON group, 6 patients were diagnosed with clinical definite MS with optic neuritis; 4 patients had presented with CIS with brain or brainstem demyelinated lesion and subsequently got optic neuritis; the other 52 patients presented with isolated acute optic neuritis fulfilling the idiopathic demyelinating etiology after thorough ancillary work-up. Among the 19 R-ON patients, the recurrent times differ from 8 to 3. Among the 37 NMO-ON patients, 5 had previous myelitis and all patients showed a seropositive for AQP4-Ab.

The mean age in NMO-ON group was significantly older than other groups (*P* = 0.01), whereas there was no difference in age between MS-ON, R-ON, and control. The mean disease duration was significantly longer in R-ON and NMO-ON groups compared to MS-ON (*P* = 0.02). R-ON group showed high prevalence of bilateral involvement than MS-ON and NMO-ON group (*P* = 0.01). There was no statistic difference in female prevalence in all groups.

### 3.2. RNFL Measurement

Because age is known to influence retinal thickness parameters, first of all, covariance was analyzed using a linear regression model and the results showed there was no significant relation between age and RNFL thickness in our groups of subjects. Peripapillary RNFL thickness measured in 3 optic neuropathy groups was significantly thinning compared to healthy controls (Table 2). After repeat measurement of variance of 4 quadrants in each group, the mean difference showed an average of RNFL loss in MS-ON, R-ON, and NMO-ON groups of 32.8 *μ*m, 46.9 *μ*m, and 43.8 *μ*m, respectively, compared to healthy control (Table 3). Furthermore, the R-ON and NMO-ON patients showed significant decreased RNFL in eyes in all quadrants compared with MS-ON, whereas there was no significant difference between R-ON and NMO-ON group.

### 3.3. Macular GCC Measurement

**Figure 1:**
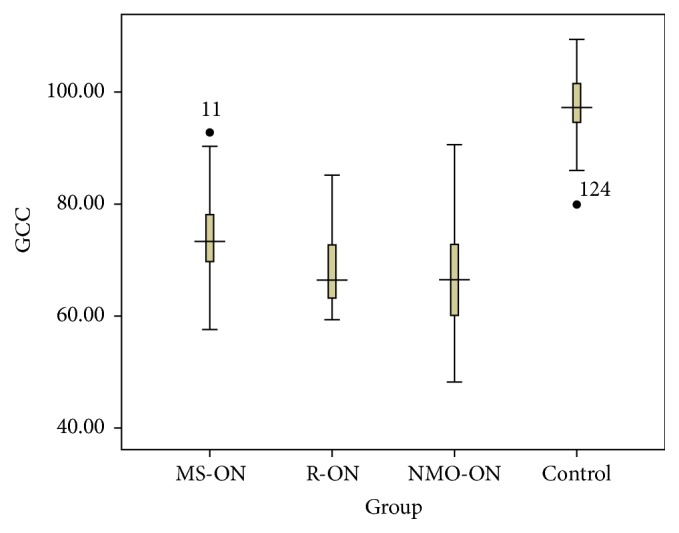
The boxplot analysis representing the macular GCC thickness in MS-ON, R-ON, NMO-ON, and control group. Mean values and 5% and 95% percentiles are shown. The difference between the 4 groups was statistically significant (*P* < 0.001), whereas there was no significant difference between R-ON and NMO-ON group (*P* = 0.725).

For the macular GCC, the tendency was the same as the peripapillary RNFL pattern, which showed significantly reduced in 3 optic neuritis groups compared to control (Figure 1). An average GCC thinning in MS-ON, R-ON, and NMO-ON groups was of 24.2 *μ*m, 28.5 *μ*m, and 28.5 *μ*m, respectively, compared to healthy control. There were no statistic differences for the GCC between R-ON and NMO-ON.

## 4. Discussion

Optic neuritis is one of the common optic neuropathies, which lead to visual loss in young Chinese and the underline etiologies have not been full clarified [1]. Our cohort study composed of a group of typical MS related optic neuritis patients, as well as atypical forms like R-ON and NMO-ON. Most of these patients presented as first attack which can be a manifestation of MS, NMO, or other unknown inflammatory disorders. Although the clinical characteristics and laboratory tests can help differentiating some of the etiology, the board spectrum of optic neuritis made it difficult to a definite diagnosis in a short term after one optic neuritis episode.

SD-OCT is a very useful and objective method to provide data on RNFL and macular GCC thickness and volumes. Also the eye tracking systems permit perfect repositioning in longitudinal studies for investigators to capture subtle changes on the order of a few micrometers.

**Figure 2:**
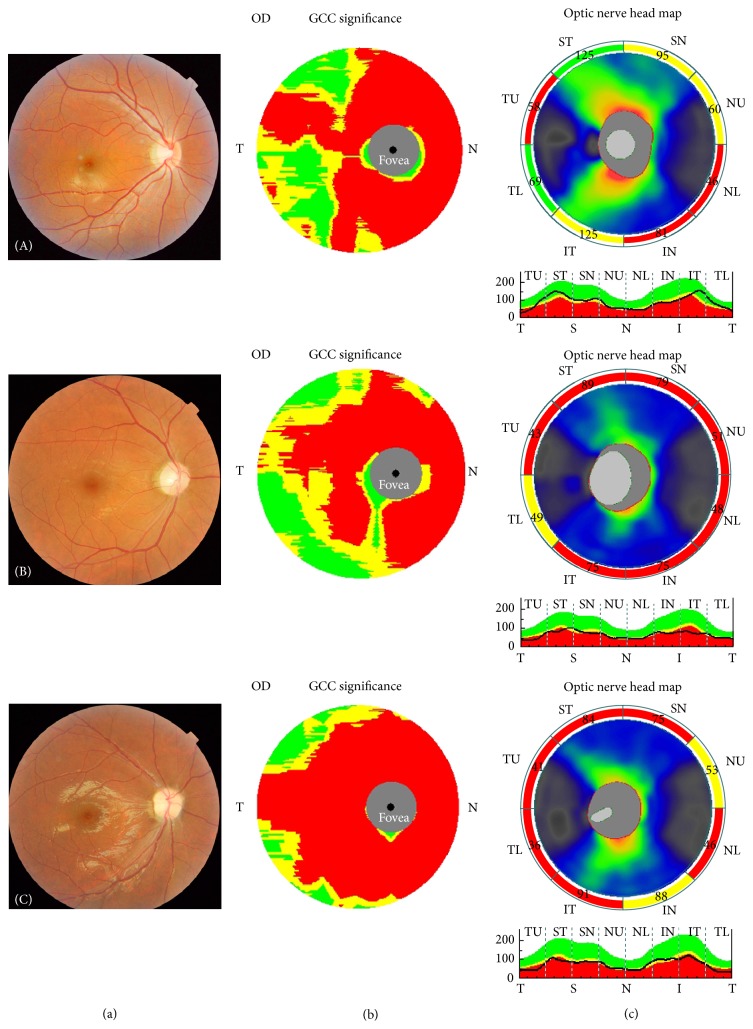
The fundus photograph (a) together with the corresponding macular GCC (b) and RNFL (c) measurements in MS-ON (A), R-ON (B), and NMO-ON (C) groups are showed, respectively.

The up to date cross-sectional studies and longitudinal investigations on OCT showed a significant alteration pattern in NMOSD patients with optic neuritis compared to MS-ON and healthy controls [18, 19]. A meta-analysis showed a loss of approximately 20 m in the affected eye in relapsing-remitting MS compared to healthy controls [20]. Bichuetti et al. [12] research also showed that, in patients with NMO and chronic relapsing inflammatory optic neuritis, the RNFL tend to have significantly lower thickness than patients with MS-ON. Our findings also demonstrate the same OCT pattern that the peripapillary RNFL and macular GCC thickness decreased significantly 6 months after once attack of optic neuritis compared to healthy controls. Furthermore, the R-ON and NMO-ON groups showed more severe damage compared to patients with MS (Figure 2). Approximately 40 *μ*m thinning of RNFL was found in NMO-ON and R-ON eyes compared to controls (approximately 30 *μ*m thinning in MS-ON). The temporal quadrant damage tendency in MS-ON was not shown in our cohort according to Naismith et al. study [11].

The GCC measured in our study by RTVue-100 protocol provided a value of combined macular ganglion cell layer and inner plexiform layer, which can help estimate the retrograde of optic nerve after damage. Six months after attack, the GCC showed an nearly 30 *μ*m thinning in NMO-ON and R-ON groups, as well as approximately 20 *μ*m in MS-ON compared to controls. The profound loss of GCC, which is closely associated with visual disability in MS [21], can also help in differentiating NMO or R-ON from MS-ON in early stage, where the true peripapillary RNFL will not be available due to the swell of optic disc.

The profound loss of peripapillary RNFL and macular GCC in R-ON group, whose pattern is similar to NMO-ON, to some extent, indicate that they share the some underline etiology. In addition, the OCT technique makes it possible to measure the single layer of ganglion cell around macular, which will give accurate thickness of the neurons. Further prospective longitudinal investigations will be needed to illustrate the change in OCT pattern as a structure marker for axonal degeneration and neuronal loss.

## Figures and Tables

**Table 1:** Demographics and clinical characteristics for MS-ON, R-ON, and NMO-ON group and control.

Group	Patients (*n*)	Age (year) (mean ± SD)	Course (month) (mean ± SD)	Bilateral%	Female%
MS-ON	62	30.47 ± 16.71	6.2 ± 3.0	33.3%	58.1%
R-ON	19	31.26 ± 11.20	20.0 ± 22.6	100%	68.4%
NMO-ON	37	40.54 ± 13.64	25.0 ± 33.4	34.7%	83.8%
Control	68	31.96 ± 13.78	NA	NA	64.7%
*P* value		*P* = 0.007^a^	*P* = 0.02^b^	*P* = 0.01^c^	*P* = 0.07^d^

NA: not applicable; a: the statistical difference between NMO-ON and other groups; b: the statistical difference between MS-ON and other ON subtypes; NMO-ON and other groups; c: the statistical difference between R-ON group and other ON subtypes; d: the statistical difference between ON subtypes and control.

**Table 2:** Peripapillary RNFL thicknesses (*μ*m) for eyes of patients in each group.

RNFL	MS-ON	R-ON	NMO-ON	Control
Average	79.12 ± 15.64	56.06 ± 9.83	63.94 ± 11.86	112.01 ± 10.93
Temporal	57.94 ± 14.57	48.37 ± 11.25	46.59 ± 12.10	139.93 ± 19.27
Superior	100.43 ± 22.51	80.74 ± 9.50	79.45 ± 16.47	120.43 ± 30.71
Nasal	59.84 ± 17.59	48.37 ± 8.90	48.91 ± 14.08	81.28 ± 13.05
Inferior	98.29 ± 21.57	82.76 ± 17.87	80.82 ± 17.40	141.76 ± 20.19

**Table 3:** Repeated measures ANOVA of multiple comparisons of each group.

(*I*) group	(*J*) group	Mean difference (*I* − *J*)	Std. error	Sig.	95% confidence interval	
					Lower bound	Upper bound
MS-ON	R-ON	14.087^*∗*^	3.558	.000	7.066	21.108
	NMO-ON	10.998^*∗*^	3.336	.001	4.415	17.581
	Control	−32.795^*∗*^	2.312	.000	−37.358	−28.232
R-ON	NMO-ON	−3.089	4.278	.471	−11.531	5.353
	Control	−46.882^*∗*^	3.547	.000	−53.881	−39.882
NMO-ON	Control	−43.793^*∗*^	3.309	.000	−50.322	−37.263

^*∗*^The mean difference is significant at the 0.05 level.
